# Emerging Trends and Research Foci in Artificial Intelligence for Retinal Diseases: Bibliometric and Visualization Study

**DOI:** 10.2196/37532

**Published:** 2022-06-14

**Authors:** Junqiang Zhao, Yi Lu, Yong Qian, Yuxin Luo, Weihua Yang

**Affiliations:** 1 Department of Medical Engineering Xinxiang Medical University Xinxiang, Henan China; 2 Department of Nursing Xinxiang Medical University Xinxiang, Henan China; 3 Jiangsu Testing and Inspection Institute for Medical Devices Nanjing, Jiangsu China; 4 The Laboratory of Artificial Intelligence and Bigdata in Ophthalmology Affiliated Eye Hospital of Nanjing Medical University Nanjing, Jiangsu China

**Keywords:** artificial intelligence, retinal disease, data visualization, bibliometric, citespace, VOSviewer, retinal, eye, visual impairment

## Abstract

**Background:**

Patients with retinal diseases may exhibit serious complications that cause severe visual impairment owing to a lack of awareness of retinal diseases and limited medical resources. Understanding how artificial intelligence (AI) is used to make predictions and perform relevant analyses is a very active area of research on retinal diseases. In this study, the relevant Science Citation Index (SCI) literature on the AI of retinal diseases published from 2012 to 2021 was integrated and analyzed.

**Objective:**

The aim of this study was to gain insights into the overall application of AI technology to the research of retinal diseases from set time and space dimensions.

**Methods:**

Citation data downloaded from the Web of Science Core Collection database for AI in retinal disease publications from January 1, 2012, to December 31, 2021, were considered for this analysis. Information retrieval was analyzed using the online analysis platforms of literature metrology: Bibliometrc, CiteSpace V, and VOSviewer.

**Results:**

A total of 197 institutions from 86 countries contributed to relevant publications; China had the largest number and researchers from University College London had the highest H-index. The reference clusters of SCI papers were clustered into 12 categories. “Deep learning” was the cluster with the widest range of cocited references. The burst keywords represented the research frontiers in 2018-2021, which were “eye disease” and “enhancement.”

**Conclusions:**

This study provides a systematic analysis method on the literature regarding AI in retinal diseases. Bibliometric analysis enabled obtaining results that were objective and comprehensive. In the future, high-quality retinal image–forming AI technology with strong stability and clinical applicability will continue to be encouraged.

## Introduction

Retinal diseases are the main afflictions affecting human vision. Diabetic retinopathy (DR) is an eye vascular disease caused by diabetes [1]. Following DR, retinal vein occlusion is the most frequent retinal vascular disorder [2]. Drusen, long-spaced collagen, and phospholipid vesicles are all linked to age-related macular degeneration (AMD). These structures exist between the retinal pigment epithelium’s basement membrane and the rest of the Bruch membrane [3]. Glaucoma is a disease that leads to the death of retinal ganglion cells as well as the loss of axons that make up the optic nerve [4]. Early detection of the disease is challenging; however, the condition may be improved with appropriate treatment [5]. These lesions are the major cause of vision loss or impairment in working-age and elderly adults worldwide [6,7]. The identification of retinopathy and maculopathy retinopathy may be time-intensive and requires expert training.

Artificial intelligence (AI), in which training data are used to develop a system, has become increasingly popular regarding clinical image analysis and disease diagnosis [8-13]. The US Food and Drug Administration has approved a device based on AI to diagnose DR, despite the fact that the application and development of AI in medicine are still in an infancy stage [14]. To address the current limitations of auxiliary examination processes, computer algorithms determine the optimal decision boundary in a multidimensional feature space [15]. At present, such systems are still being improved by researchers.

The aim of this study was to gain insights into the overall application of AI technology in the research of retinal diseases from specific time and space dimensions. We used bibliometric methods to analyze papers in the Science Citation Index (SCI) reporting studies performed from January 1, 2012, to December 31, 2021, on AI in retinal disease research. The citations of countries, regions, institutions, periodicals, study categories, keywords, and references were included in the data. In addition, we established a visual and unbiased approach to explore hotspot knowledge frontiers in a research area. This study thus provides a useful reference for algorithm researchers, ophthalmologists, and experts in the field of medical engineering.

## Methods

### Paper Selection

**Figure 1:**
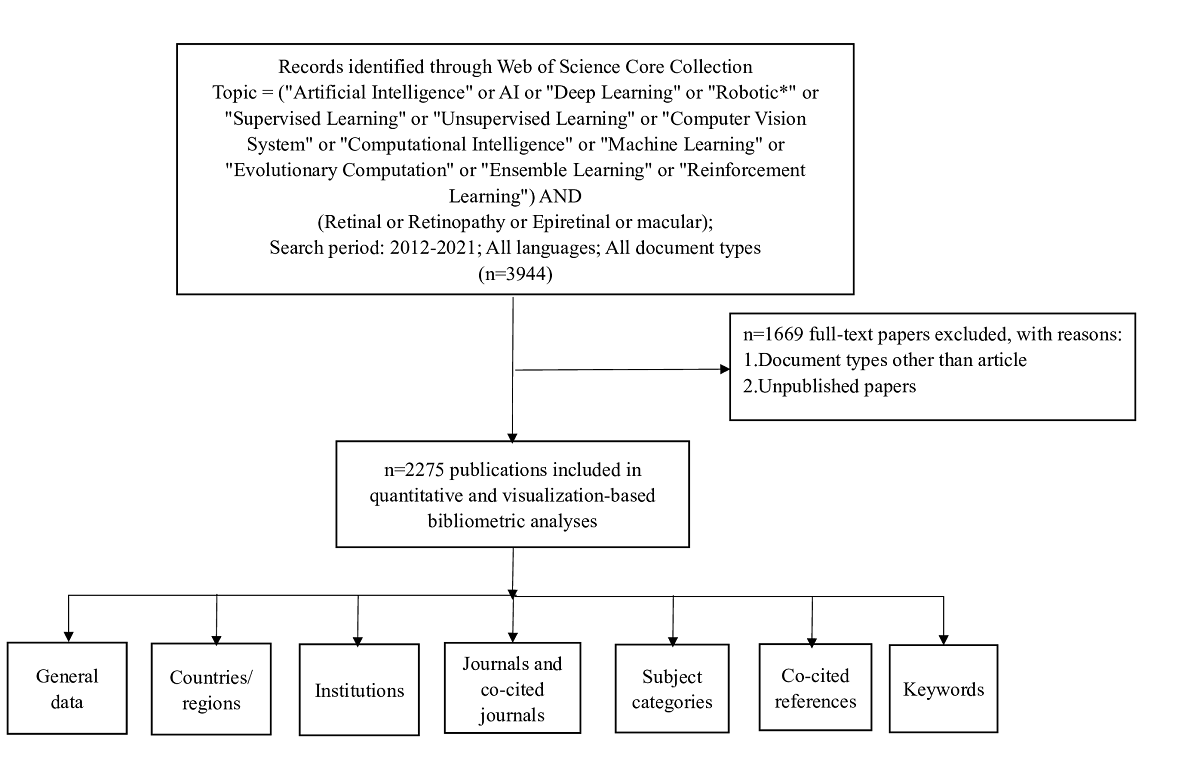
Frame flow diagram for the detailed selection criteria and bibliometric analysis steps of applying artificial intelligence (AI) to the study of retinal diseases in the Web of Science Core Collection database.

On February 15, 2022, all citation data published between January 1, 2012, and December 31, 2021, were retrieved from the Web of Science Core Collection (WoSCC). The data were independently verified by two authors (YL and JZ). The detailed search string is listed in Figure 1. The document type was article. From each publication, we gathered the following basic data: title, abstract, authors, institution, country or region, journal, keywords, and references. The detailed search and analysis processes are depicted in Figure 1.

### Data Exclusion

Unpublished and document types other than articles were excluded. The citation data were downloaded on February 15, 2022, and some 2021 documents included by WoSCC were not published and were thus not included in this study. Some data were excluded because their document types were not articles, such as procedures, papers, review articles, meeting abstracts, early access, editorial materials, book chapters, letters, corrections, data papers, books, and retracted publications.

### Statistical Analysis

Collaborative networks of countries, institutions, journals, keywords, and research categories were analyzed and visualized using the bibliometrics online analysis platform Bibliometrc [16], CiteSpace V, and VOSviewer. We collected detailed citation features for analysis, including the number of annual publications, countries, institutes, journals, subject categories, cocited references, and keywords. The H-index represents an estimate of the importance and general impact of the research contributions [17].

## Results

### Distribution of Articles by Publication Year

**Figure 2:**
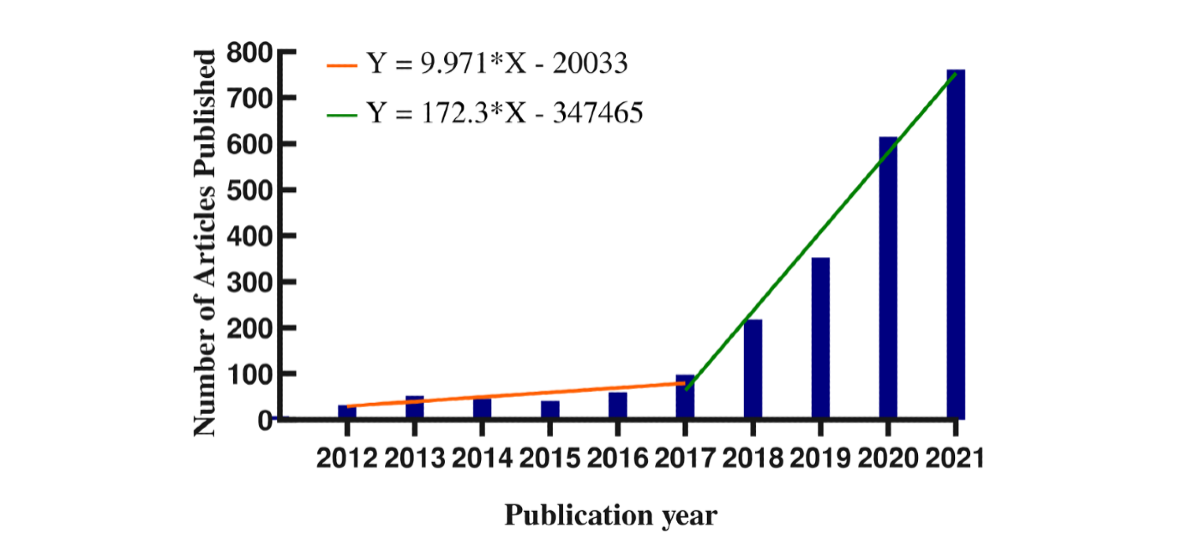
Trends in the number of publications on applying artificial intelligence to the study of retinal diseases from 2012 to 2021.

We analyzed a total of 2275 papers published between 2012 and 2021. The numbers of published studies on the application of AI technology to retinal illnesses over time are summarized in Figure 2. Since 2017, the number of studies on the use of AI in the treatment of retinal illnesses has skyrocketed.

### Countries or Regions and Institutes

**Figure 3:**
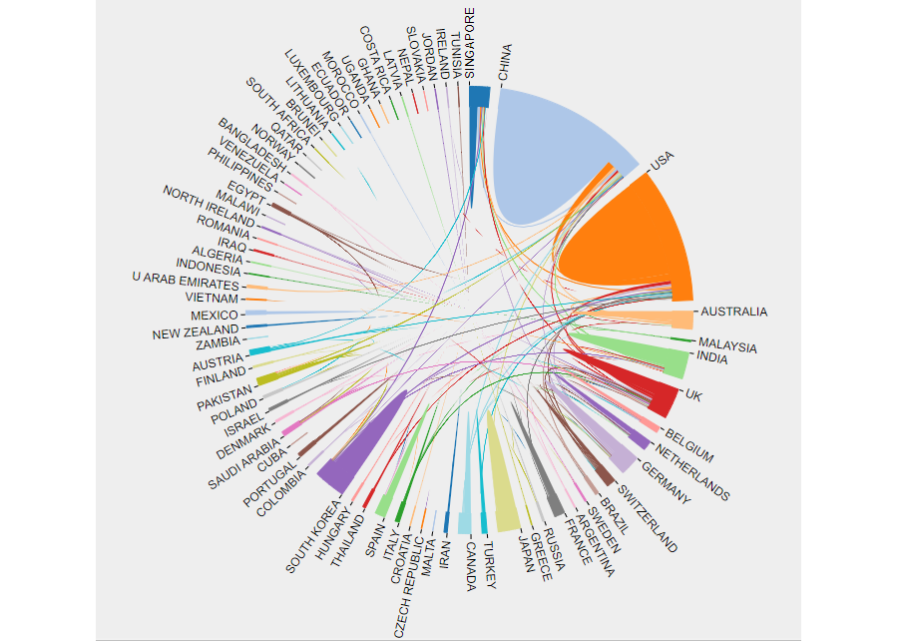
The cooperation of countries or regions that contributed to publications on applying artificial intelligence to the study of retinal diseases from 2012 to 2021.

**Figure 4:**
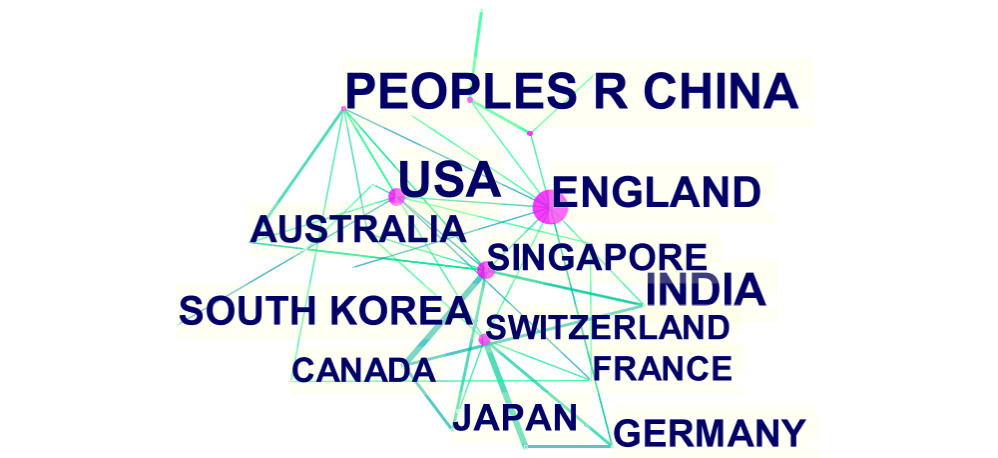
The cooperation of countries or regions that contributed to publications on applying artificial intelligence to the study of retinal diseases from 2012 to 2021.

These citations mentioned a total of 86 nations or territories. In Figure 3, the publications from different countries or regions are represented by blocks of different colors. The size of the colored block area represents the number of citations and the size of the different colored coverage areas represents the intensity of the cooperation. In Figure 4, a larger label area for a given country represents a greater contribution to the related literature. The purple node area indicates the strength of the centrality; the higher the centrality value, the more cooperative the relations it establishes in the country where the node is located. As per Figures 3 and 4, China and the United States have contributed the largest number of documents to this field. The United States, the United Kingdom, and Singapore cooperated more with other countries.

Table 1 lists the top 10 countries cited. China had the largest number of publications, followed by the United States, India, and the United Kingdom. Britain had the strongest centrality, followed by the United States and Singapore.

**Figure 5:**
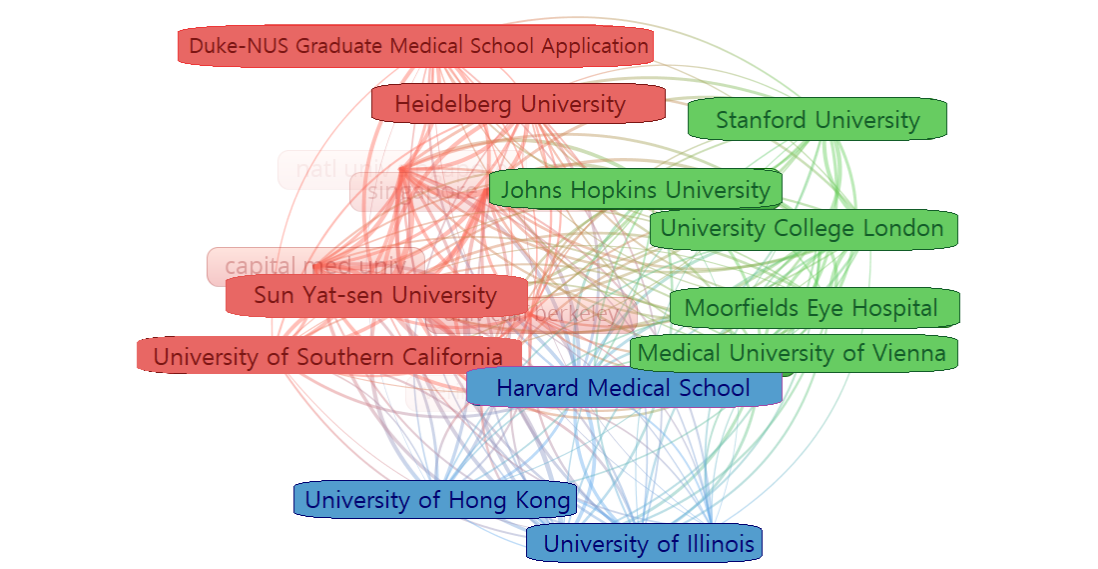
Network map of institutions that contributed to publications on the application of artificial intelligence in retinal diseases from 2012 to 2021.

A total of 197 institutions have published relevant papers, and the clustering of their cooperative relationships is shown in Figure 5. The top 10 institutions regarding the frequency of cited institutions are listed in Table 1, including three Chinese institutions (Sun Yat Sen University, Chinese Academy of Sciences, and Shanghai Jiao Tong University), three US institutions (Johns Hopkins University, Oregon Health and Science University, and Stanford University), two Singapore institutions (Singapore National Eye Centre and National University Singapore), one Austrian institution (Medical University of Vienna), and one UK institution (University College London). Among them, the number of citations with authors from University College London ranked in 10th position; however, their H-index was the highest. In addition, Johns Hopkins University and University College London, which were the two highest-ranked institutions in the center, appeared in the same cluster.

**Table 1:** The top 10 countries or regions and institutions with publications on the application of artificial intelligence in retinal diseases from 2012 to 2021.

Rank		Count	Centrality	H-index
**Country or region**				
	1. People’s Republic of China	634	0.16	42
	2. United States	620	0.57	56
	3. India	309	0.04	33
	4. England	205	1.00	33
	5. South Korea	150	0.00	24
	6. Germany	132	0.04	24
	7. Australia	120	0.00	30
	8. Japan	98	0.00	19
	9. Singapore	98	0.49	20
	10. Canada	74	0.06	19
**Institution**				
	1. Sun Yat Sen University	62	0.05	16
	2. Chinese Academy of Science	51	008	14
	3. Johns Hopkins University	49	0.13	16
	4. Oregon Health and Science University	48	0.01	16
	5. Stanford University	47	0.03	18
	6. Medical University of Vienna	42	0.06	19
	7. Singapore National Eye Centre	39	0.11	18
	8. National University of Singapore	38	0.07	20
	9. Shanghai Jiao Tong University	38	0.00	10
	10. University College London	37	0.12	21

### Journals

**Figure 6:**
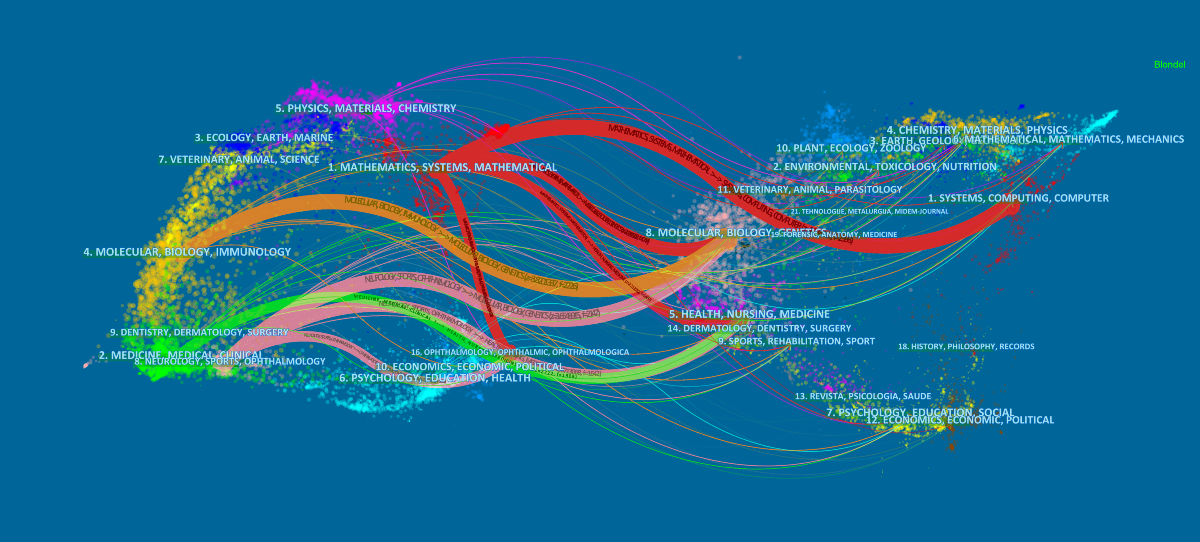
The dual-map overlay of journals that contributed to publications on the application of artificial intelligence in retinal diseases from 2012 to 2021. Red represents the greatest influence.

Citing journals represents the research frontier and cited journals represents the research foundation. The top 10 citing journals and cited journals are shown in Table 2. The most frequently cited journals in the included citations were *Translational Vision Science Technology*, *Scientific Reports*, and *IEEE Access*. The journals that appeared most frequently among the cited journals were *Ophthalmology*, *British Journal of Ophthalmology*, and *IEEE Transactions on Medical Imaging*. *PLoS One* appeared in both the top-ranked citing and cited journals lists. The dual map of the journals is shown in Figure 6. Red represents the discipline field with the greatest influence. The research influence in the field of mathematics/systems/mathematical subject ranked first among the citing journals.

**Table 2:** The top 10 citing journals and cited journals of publications on the application of artificial intelligence in retinal diseases from 2012 to 2021.

Rank		Count
**Citing journals**		
	1. Translational Vision Science Technology	100
	2. Scientific Reports	86
	3. IEEE Access	85
	4. Biomedical Optics Express	67
	5. PLoS One	53
	6. IEEE Transactions on Medical Imaging	48
	7. American Journal of Ophthalmology	41
	8. Computer Methods and Programs in Biomedicine	40
	9. British Journal of Ophthalmology	36
	10. Eye	31
**Cited journals**		
	1. Ophthalmology	1140
	2. Investigative Ophthalmology & Visual Science	1083
	3. IEEE Transactions on Medical Imaging	974
	4. Lecture Notes in Computer Science	855
	5. British Journal of Ophthalmology	778
	6. PLoS One	775
	7. Medical Image Analysis	714
	8. JAMA (Journal of the American Medical Association)	681
	9. American Journal of Ophthalmology	673
	10. JAMA Ophthalmology	647

### Research Category

**Figure 7:**
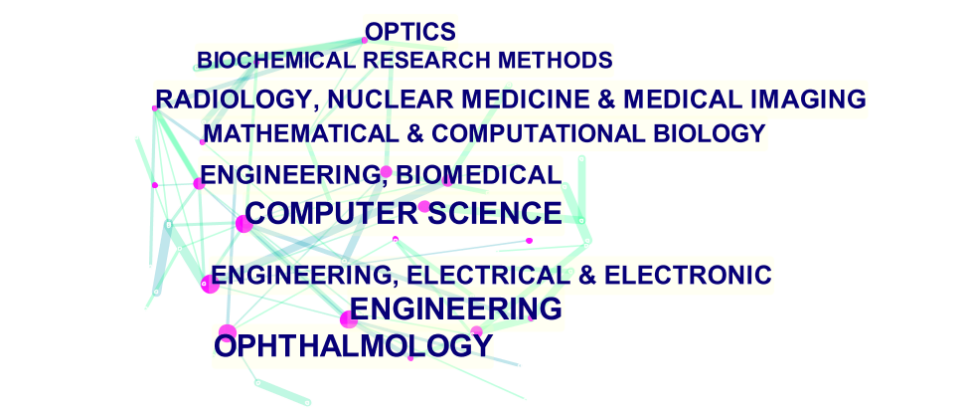
Network map of the research categories of publications on the application of artificial intelligence in retinal diseases from 2012 to 2021.

Figure 7 and Table 3 present the research areas of the citations. The most involved research areas were Ophthalmology and Engineering Electrical Electronic. The highest H-index score areas were Engineering Biomedical and Radiology Nuclear Medicine Medical Imaging. This indicates that research on AI in retinal diseases is primarily focused within the fields of computer engineering and medical imaging.

**Table 3:** The top 10 research categories of publications on the application of artificial intelligence in retinal diseases from 2012 to 2021.

Rank	Research category	Count	H-index
1	Ophthalmology	489	40
2	Engineering Electrical Electronic	332	33
3	Engineering Biomedical	318	42
4	Computer Science Artificial Intelligence	265	34
5	Computer Science Interdisciplinary Applications	250	40
6	Radiology Nuclear Medicine Medical Imaging	246	42
7	Computer Science Information Systems	206	23
8	Multidisciplinary Sciences	185	25
9	Medical Informatics	173	30
10	Mathematical Computational Biology	139	24

### Keywords

**Figure 8:**
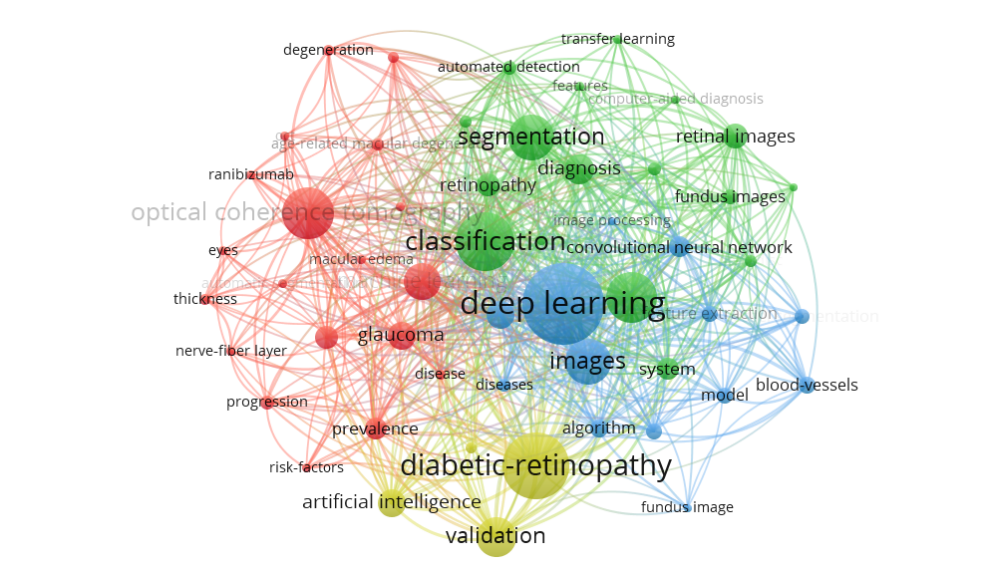
Network map of the 50 top-ranking keywords divided into four clusters.

**Figure 9:**
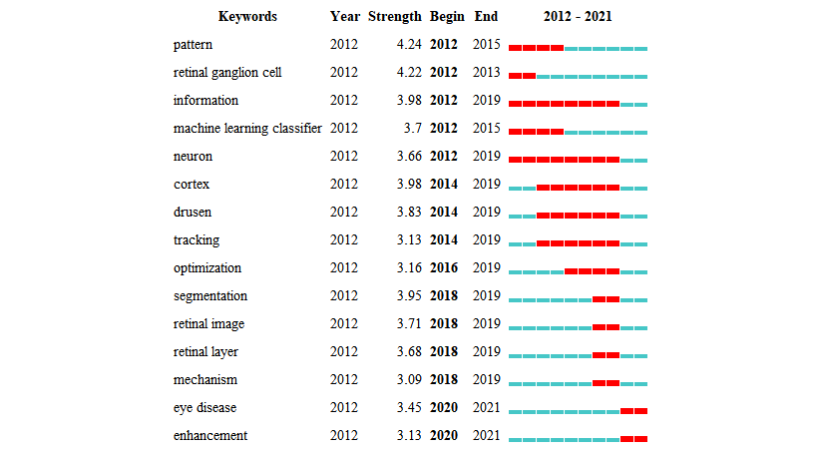
Top 15 keywords with the strongest citation bursts of publications on the application of artificial intelligence in retinal diseases from 2012 to 2021. Red indicates the emergence of keywords.

Keywords were retrieved and examined from the relevant literature. Table 4 lists the top 20 keywords used. Among them, the keywords cited over 200 times were “diabetic retinopathy,” “classification,” “validation,” and “imaging.” The keywords of the 2267 articles were analyzed and divided into four categories (deep learning, DR, optical coherence tomography, and classification), as shown in Figure 8. The time trend was examined using the hotspot transfer method, which was applied to the first 15 keywords with the highest citation outbreak. As shown in Figure 9, the key words with the greatest outburst intensity were “pattern” and “retinal ganglion cell.” The red grid indicates the emergence of keywords. “Information” and “neuron” were the keywords with the longest use (2012-2019), and “eye disease” and “enhancement” were the most popular keywords from 2018 to 2021.

**Table 4:** The top 20 keywords on the application of artificial intelligence in retinal diseases from 2012 to 2021.

Rank	Keyword	Count
1	Diabetic retinopathy	380
2	Classification	271
3	Image	270
4	Validation	224
5	Segmentation	213
6	Optical coherence tomography	154
7	Diagnosis	152
8	System	133
9	Prevalence	124
10	Macular degeneration	118
11	Algorithm	111
12	Retinal image	110
13	Disease	106
14	Model	105
15	Neural network	104
16	Blood vessel	103
17	Retinopathy	101
18	Eye	99
19	Progression	82
20	Automated detection	77

### Reference Network

**Figure 10:**
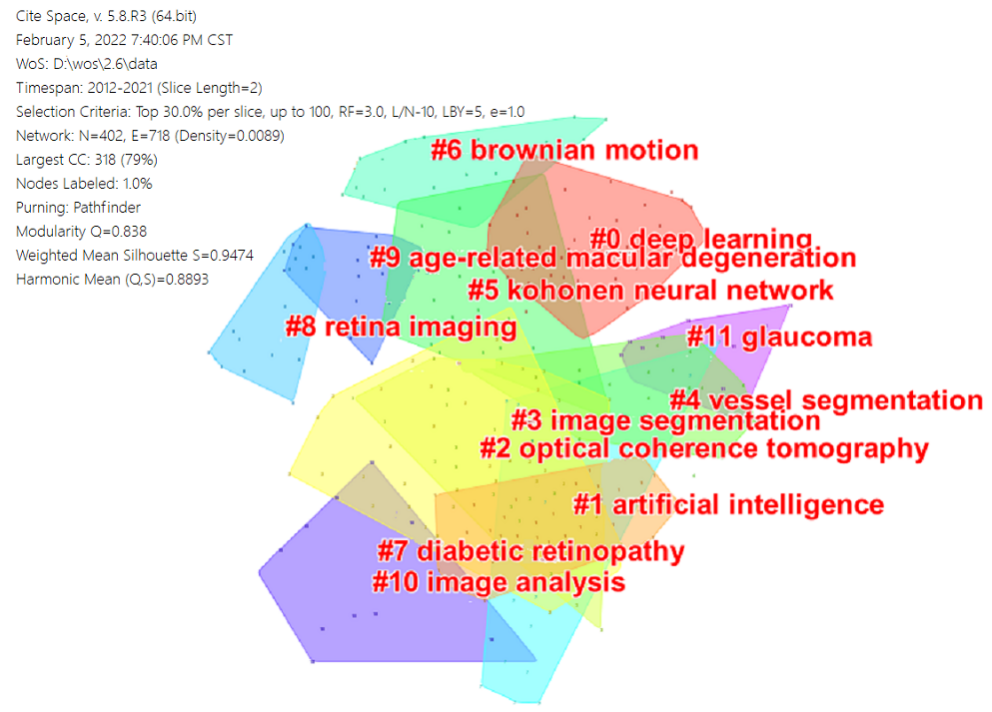
Reference cocitation map of publications on the application of artificial intelligence in retinal diseases from 2012 to 2021.

A reference’s citation frequency represents its influence. We constructed a cocited literature network and analyzed the scientific relevance of the publications. The modularity of the network was measured using the modularity index; the higher the modularity Q score, the better the network’s clustering. Q>0.3 indicates that the network community structure obtained is noteworthy. The better the network’s homogeneity, the closer the Silhouette S value is to 1. The cited documents were grouped into 12 clusters, as shown in Figure 10. The homogeneity of these clusters was characterized by modularity Q=0.838 and weighted mean Silhouette S=0.9474. Cluster markers were created using the index items taken from the literature. The modularity of the network was measured using the modularity index. “Deep learning“ was the cluster with the widest range.

### Overall Results

There were 2275 SCI papers reviewed in this study of AI and retinal diseases, which were published from 2012 to 2021, with a major increase in these publications since 2017. This is due to one study [18] being cited 506 times in 2016, which provided an important scientific and technical reference for researchers in this field. China and the United States had the maximum number of publications. The United Kingdom had the most cooperative relations. In addition, the three outcome indicators of the institution indicated that the research conducted by University College London and Johns Hopkins University has had a major influence on this research field. The number of citations in this field published by citing journals and cited journals indicated that the application of AI in retinal diseases has mainly been in the fields of computer engineering and medical imaging based on digital technology. This same pattern also applied to the citation categories.

The important nodes in the clustering demonstrated this research field’s knowledge bases after clustering the cited references. They were labeled #0 deep learning, #1 artificial intelligence, #2 optical coherence tomography, #3 image segmentation, #4 vessel segmentation, #5 Kohonen neural network, #6 Brownian motion, #7 diabetic retinopathy, #8 retina imaging, #9 age-related macular degeneration, #10 image analysis, and #11 glaucoma. The top 10 cocited references in these clusters are listed in Table 5. These studies’ conclusions require a substantial amount of basic work to be performed by ophthalmologists. For example, from the top-ranking article, 54 ophthalmologists or ophthalmic trainees participated in the identification of 5-year image data of three eye hospital patients presenting for image-based DR screening. From the second-ranked article, 494,661 retinal photographs were used to assess the diagnosis performance of a deep-learning system for DR and related eye illnesses. Each retinal image was analyzed by two trained senior, certified nonmedical professional graders. To achieve progress in the application of AI in retinal diseases, more cooperative and collaborative relationships among ophthalmologists, imaging technicians, and computer technology researchers are required in the future.

**Table 5:** The top 10 publications on the application of artificial intelligence (AI) in retinal diseases from 2012 to 2021.

Rank	Reference	Title of cocited reference	Count	Interpretation of the findings
1	Gulshan et al [18]	Development and validation of a deep learning algorithm for the detection of DR^a^ in retinal fundus photographs	506	For diagnosing referable DR, a deep machine learning method had good sensitivity and specificity
2	Ting et al [19]	Development and validation of a deep learning system for DR and related eye diseases using retinal images from multiethnic populations with diabetes	270	The deep learning system demonstrated high sensitivity and specificity for detecting DR and related eye disorders
3	Lam et al [20]	Automated Identification of DR using deep learning	214	A high-reliability data-driven AI-based grading technique for screening and identifying fundus pictures taken from patients with diabetes. For further assessment and therapy, these patients should be referred to an ophthalmologist
4	Ronneberger et al [21]	U-Net: convolutional networks for biomedical image segmentation	208	This research provides a network and training technique that heavily depends on data augmentation to make better use of existing annotated samples
5	He et al [22]	Deep residual learning for image recognition	173	This research proposes a residual learning paradigm for network training
6	Kermany et al [23]	Identifying medical diagnoses and treatable diseases by image-based deep learning	163	This paper describes the development of a diagnostic tool for screening patients with common treatable blinding retinal disorders based on a deep-learning architecture
7	LeCun et al [24]	Deep learning	162	AI will advance as a result of systems that combine representation learning and complicated reasoning
8	De Fauw et al [25]	Clinically applicable deep learning for diagnosis and referral in retinal disease	160	When using tissue segmentations from a different type of device, a unique deep learning architecture was used to a clinically diverse data set to retain referral accuracy
9	Abràmoff et al [26]	Improved automated detection of DR on a publicly available dataset through the integration of deep learning	159	Deep learning–enhanced algorithms have the potential to improve the effectiveness of DR screening, thereby preventing vision loss and blindness from this dreadful disease
10	Esteva et al [27]	Dermatologist-level classification of skin cancer with deep neural networks	145	This research shows how deep learning works in dermatology and how it can be applied to other fields, including ophthalmology, otolaryngology, radiography, and pathology

^a^DR: diabetic retinopathy.

## Discussion

### Research Hotspots and Frontiers

#### Overview

Keywords provide a quick summary of the most important aspects and points in a collection of articles [28]. Current research hotspots and frontiers can be identified using burst keyword analysis. Following the capture of the burst keywords, two study fields were identified: eye disease (2018-2021) and enhancement (2018-2021).

#### Eye Disease

The application of AI technology to eye diseases is comparable to the best clinical systems and has achieved competitive results in solving issues related to the diagnosis and monitoring of complex ophthalmic diseases. AMD is usually asymptomatic, and an intermediate stage may not be identified. Moreover, AMD affects several people worldwide and thus identifying it can be time-consuming and difficult without the assistance of experts. Fortunately, applying deep learning–based automated algorithms may solve this challenge. This could also address the expenses of screening or monitoring, health care access, and the evaluation of innovative treatments for AMD development or progression [29]. DR also causes challenges for many people. It is the leading cause of vision loss and preventable blindness in adults aged 20-74 years in middle- and high-income countries [30]. Using a combination of digital retinal image analysis and telemedicine assessment to help identify people at risk of cardiovascular disease and cognitive impairment may have benefits beyond sight-threatening diseases prevention [11,31]. Aamir et al [32] built a hierarchical deep convolutional neural network (CNN) for glaucoma recognition and prevention using an advanced deep-learning technique. This project was then used to extract multilayer features from 1338 images to verify the performance of the algorithm, achieving nearly 100% specificity, sensitivity, accuracy, and precision. These studies highlight the current research findings based on the use of AI technology in clinical applications for the management of ophthalmic diseases.

In addition, AI technology has been applied to the screening, referral, diagnosis, health care, and follow-up visits of patients with a variety retinal illness. Wang et al [33] developed a two-step semiautomatic deep learning algorithm–assisted technique to identify fundus pictures and aid in the detection of DR with vision-threatening complications. Optical coherence tomographic (OCT) angiography is a noninvasive imaging technology that may generate angiograms at precise depths within the retina as well as visualize the microvasculature in real time [34]. A British study published in 2021 assessed an AI decision support system with the use of OCT scanning of retinal pictures to identify the digital referral path, providing evidence of the contributing reasons and difficulties of adopting the digital path in real life, with the goal of helping to eliminate unnecessary referrals [35]. To boost doctors’ faith in AI systems to make accurate diagnoses, some AI systems must be written as interpretable programs [36]. According to a follow-up poll conducted in 2017, radiologists used certain touch-environment solutions forced by the clinical setting at the time, demonstrating that they are still opposed to the transfer from traditional to updated interfaces [37]. 

#### Enhancement

Ophthalmologists are confused by the quality differences among fundus diagnostic images [38]. Enhancing the analysis of retinal image structures requires the development of a computer-assisted algorithm to correct the low fundus image quality [39]. Wan et al [40] proposed a deep learning–based technique that overcomes the limitations of current imaging algorithms and improves the low retinal image quality. CNN models may be developed under strong and mostly correct assumptions regarding the nature of macular disease images [41]. El-Hag et al [42]established the importance of the proposed blurry image improvement phase. Additionally, using CNN as a classification technique with hazy logic augmentation was shown to improve the classification of normal and abnormal outcomes. In the testing phase, this resulted in a classification accuracy of 100% [42]. The blood vessels in the neural network must be divided into arteries and veins to diagnose hypertensive retinopathy using retinal diagnostic images. According to this demand, Hussein and Faheem [43] proposed the use of an AI method to improve vascular contrast. Zhou et al [44] proposed the learning of discriminative CNN features and enhanced thin vessels in color fundus images to further improve the segmentation performance. This algorithm improves the contrast of the retinal vessels and was verified by three pediatric ophthalmologists [45]. Goel et al [46] showed that using a development learning model to transfer learning can improve the accuracy of correct classification of different aneurysms in the retina area caused by DR.

AI technology can improve diagnosis accuracy and can also save time for both doctors and patients by increasing the contrast between image structures, such as segmenting distinct blood arteries or calculating normal and pathological structures. The image quality of retinal disease examination needs to be unified with high precision. This is currently a research hotspot to provide more high-quality research images based on research and development in AI technology, improvements in image acquisition technology, and the standardization of acquisition steps.

### Limitations

This bibliometric analysis only included the literature data in WoSCC. Some other databases were not included, such as PubMed, Medline, and Cochrane. In addition, the citation data analyzed were only from the literature published from 2012 to 2021, rather than collecting all articles published in this research field to date. Some 2022 studies are still ongoing and have not yet been published. These criteria may result in publication bias.

### Conclusions

This study provides a systematic literature analysis on the use of AI in retinal diseases. Bibliometric analysis enabled obtaining objective and comprehensive results. Judging by the volume of published papers and research subjects, this study area is still popular and a noteworthy topic with major interdisciplinary exploration space. Ophthalmologists, imaging experts, and computer algorithm researchers in developing and developed countries need to make full use of population advantages or core technologies in different regions to strengthen collaboration. This idea has become a research hotspot that uses the existing basic clinical research results and a more advanced algorithm mode to develop a high-quality ophthalmic examination image system and further verify its clinical applicability. At present, an algorithm program with 100% diagnostic accuracy for retinal disease has been developed [42]. In the future, high-quality retinal image–forming AI technology with strong stability and clinical applicability will continue to be encouraged.

## Abbreviations

AI: artificial intelligence
AMD: age-related macular degeneration
CNN: convolutional neural network
DR: diabetic retinopathy
OCT: optical coherence tomography
SCI: Science Citation Index
WoSCC: Web of Science Core Collection
